# Lower Extremity Orthopedic Augmentation Utilizing a Synthetic Hybrid-Scale Fiber Matrix

**DOI:** 10.7759/cureus.45046

**Published:** 2023-09-11

**Authors:** Calvin J Rushing

**Affiliations:** 1 Surgery, Baylor Scott & White Medical Center – Sunnyvale, Sunnyvale, USA

**Keywords:** case series, fracture, orthopedics, lower extremity, reconstructive surgery, wound healing, synthetic hybrid-scale matrix, extracellular matrix

## Abstract

Introduction

Lower extremity injuries represent about 15% of all emergency room visits in the United States, with ankle injuries accounting for 20% of these. Surgical site infections resulting from ankle reconstructive surgery can result in severe complications, including amputation. Depending on the procedure performed, surgical site infections of ankle reconstructive surgery can occur at a rate as high as 14%. A synthetic hybrid-scale fiber matrix with an engineered design resembling a native human extracellular matrix could be utilized to augment these difficult surgical procedures.

Materials and methods

A retrospective review of 10 patients who underwent orthopedic reconstructive surgeries of lower extremity injuries that were augmented with the synthetic hybrid-scale fiber matrix between October 2021 and February 2022 was conducted.

Results

Injuries included Achilles ruptures, bimalleolar and trimalleolar ankle fractures, ankle arthrodesis, and injuries of various other etiologies. All surgical wounds achieved complete closure and patients went on to fully recover. The average time to wound closure was 12.6 days after an average of 1.2 applications of the synthetic hybrid-scale fiber matrix. Post-reconstruction, two patients went on to have complications unrelated to the synthetic hybrid-scale fiber matrix, which then went on to heal uneventfully.

Conclusions

The positive results seen with the retrospective case series indicated that the synthetic hybrid-scale fiber matrix can promote healing and should be considered as an option in at-risk lower extremity reconstructive procedures.

## Introduction

Lower extremity injuries represent about 15% of all emergency room visits in the United States [1]. Ankle injuries accounted for 20% of these [1]. Ankle injuries including bimalleolar and trimalleolar ankle fractures are often treated surgically with an open reduction and internal fixation technique [2]. Surgical site infections resulting from this procedure range from 1.4% to as high as 14% and can result in severe complications including amputation [2-4]. The risk of surgical site infections increases in patients with multiple or severe comorbidities [3]. Patients with a history of prior surgical site and skin infections are also at a higher risk for developing these infections in subsequent procedures [5]. Other orthopedic reconstructive surgeries, such as Achilles repair surgeries, also have high rates of complications, ranging from 8% to 9.7% [6].

Augmentation of these procedures utilizing acellular matrices has been explored in prior studies [7]. These matrices are often biologic in nature and consist of collagen as well as extracellular matrix proteins [8]. Collagen, while it does support cellular ingrowth, can negatively affect cellular proliferation as it degrades [8]. Acellular matrices developed from porcine small intestine mucosa have resulted in postoperative inflammation and high complication rates when utilized in rotator cuff repairs [9]. Prior studies utilizing matrices in orthopedic augmentations have shown that synthetic grafts tend to be mechanically stronger than biologics, as well as more consistent in quality [9]. 

Given these complications, novel technology is needed in order to mitigate the risk of postoperative wound complications. This is critically important in high-risk patient populations or procedures where incision placement involves angiosomes with a precarious or tenuous blood supply. A synthetic hybrid-scale fiber matrix, similar in both size and structure to native human extracellular matrix, has shown promise in treating surgical wounds and chronic ulcers, with healing rates seen ranging from 75% to 96% [10-12]. The synthetic nature of the matrix minimizes inflammatory response and risk of infection, while also providing scaffold for cellular ingrowth and revascularization [8]. The matrix is electrospun from two synthetic, bioresorbable polymers, polyglactin 910 and polydioxanone, and engineered to resorb via hydrolysis at a rate that emulates that of cellular ingrowth [8]. As the matrix resorbs, the porosity of its structure increases, allowing for continued neovascularization and cellular differentiation [8]. The author hypothesizes that by encouraging cellular ingrowth into the surgical wound site, risk of complications associated with delayed healing may be significantly reduced. The present retrospective case series investigates the use of the synthetic hybrid-scale fiber matrix in the augmentation of orthopedic reconstructive surgeries with increased risk due to either patient co-morbidities and medical history, or the nature of the procedures itself. 

## Materials and methods

A retrospective review of patients who underwent orthopedic surgery for a lower extremity injury in which the synthetic hybrid-scale fiber matrix was utilized was conducted via a review of patient charts. Patients who received at least one application of the synthetic hybrid-scale fiber matrix at Dallas Orthopedic and Shoulder Institute in Sunnyvale, Texas between October 2021 and February 2022 to augment a lower extremity orthopedic procedure were included in this study. In each case, the patient underwent radiographic examination to determine the extent and etiology of the injury. Upon diagnosis, each patient was taken to the operating room for the clinically indicated reconstructive surgery.

Use of the synthetic hybrid-scale fiber matrix in the reconstructive procedure was determined by the surgeon based on the etiology of the injury and prior patient history. Patients with a prior history of surgical site infection or dehiscence, procedures with a high risk of dehiscence, and those with skin injury or necrosis following trauma were selected for use of the hybrid-scale fiber matrix. In each case, the synthetic hybrid-scale fiber matrix was applied to the surgical wound bed to protect hardware in the case of dehiscence, or it was placed over the wound bed to allow for re-epithelization over the open wounds resulting from prior surgical dehiscence or trauma. The synthetic hybrid-scale fiber matrix was secured within the wound bed using resorbable sutures. Surgical wounds were then sutured closed with either non-resorbable sutures or staples and dressed using nonadherent petroleum-impregnated dressings soaked in betadine, 4 x 4 gauze, cotton undercast padding, and an elastic 6” bandage with clips. All patients were instructed to wear a splint for offloading and stabilization.

## Results

A total of 10 patients were included in this retrospective case series. All 10 patients treated with the synthetic hybrid-scale fiber matrix during orthopedic reconstruction surgeries went on to fully heal. The average patient age was 52. The patients included in this study also had multiple co-morbidities including diabetes mellitus, obesity, aortic stenosis, cervical cancer, hypertension, hyperlipidemia, neuropathy, cellulitis, osteoporosis, and autoimmune disease.

Injuries included an Achilles rupture (10%), bimalleolar and trimalleolar ankle fractures (40%), ankle arthrodesis (10%), a heel wound (10%) a dorsal foot wound (10%) a pressure injury (10%), and an adult acquired flatfoot deformity (AAFD) revision (10%). All the wounds resulting from surgery achieved complete closure. Six wounds were closed at the time of surgery, and four wounds went on to heal after surgery. The average time to wound closure was 12.6 days after an average of 1.2 applications of the synthetic hybrid-scale fiber matrix (Table 1). 

**Table 1:** Patient demographics and primary healing *AAFD: Adult acquired flatfoot deformity

	Total wounds (n=10 patients)
		Patient age, mean ± SD (years)	52 ± 23
Sex (%)	
Male	30%
Female	70%
Wound etiologies	Achilles rupture (1)
	Bimal ankle fracture (3)
	Trimal ankle fracture (1)
	Heel wound (1)
	Dorsal foot wound (1)
	AAFD* revision (1)
	Pressure Injury (1)
	Ankle fusion (1)
Primary healing (%)	100%
Time to heal, mean ± SD (days)	12.6 ± 19.6
Number of synthetic hybrid-scale fiber matrix applications per case, mean	1.2
Complications	(1) Foreign body reaction to plate; (2) surgical site infection

Postreconstruction, two patients went on to have complications unrelated to the synthetic hybrid-scale fiber matrix. One patient had a foreign body reaction to a plate, and another developed a surgical site infection. In both cases, the synthetic hybrid-scale fiber matrix prevented hardware and bone exposure. Individual patient procedures and outcomes can be seen in Table 2.

**Table 2:** Individual patient outcomes *ORIF: Open reduction and internal fixation; **ATLAS: total ankle arthroplasty with anatomic lateral ankle stabilization; ***MTPJ: first metatarsal phalangeal joint

	Case 1	Case 2	Case 3	Case 4	Case 5	Case 6	Case 7	Case 8	Case 9	Case 10
Age (at time of surgery, years)	38	15	44	34	82	76	84	27	67	51
Etiology of Injury	Achilles Rupture	Trimal Ankle Fracture	Bimal Ankle Fracture	Bimal Ankle Fracture	Post-traumatic Ankle Arthritis	Heel Wound	Exposed Fibula with Osteomyelitis secondary to pressure injury	Medial Malleolus Fracture, Syndesmosis disruption, dorsal foot wound	Delayed wound healing from prior adult acquired flatfoot deformity reconstruction revision	Bimal Ankle Fracture
Operative Procedure Performed	Acute Achilles rupture repair with synthetic hybrid-scale fiber matrix application	Ankle scope with ORIF* of trimal ankle fracture and syndesmosis, deltoid ligament repair, and synthetic hybrid-scale fiber matrix application	ORIF* of bimal ankle fracture, ATLAS** of lateral ankle complex, application of synthetic hybrid-scale fiber matrix	ORIF* of bimal ankle fracture and syndesmosis with application of synthetic hybrid-scale fiber matrix	Ankle arthrodesis, incision and drainage, application of synthetic hybrid-scale fiber matrix	Incision and drainage, heel wound closure, and application of synthetic hybrid-scale fiber matrix	Staged intervention with tibiotalocalcaneal nailing and synthetic hybrid-scale fiber matrix	Ankle Scope, ORIF* for medial malleolus, ORIF* for syndesmosis, application of synthetic hybrid-scale fiber matrix to dorsal foot wound	Adult acquired flatfoot deformity reconstruction with correction of 1st MTPJ*** malunion and plantar plate repair of 2nd MTPJ***. Synthetic hybrid-scale fiber matrix applied to the wound postoperatively	staged intervention with frame application and application of synthetic hybrid-scale fiber matrix
Number of synthetic matrix pieces used	1	2	1	1	1	1	1	1	1	1
Wound Closure	Yes	Yes	Yes	Yes	Yes	Yes	Yes	Yes	Yes	Yes
Time to heal (days)	0	0	0	0	0	0	6	30	60	30
Major wound complications	no	Yes	Yes	No	No	No	No	No	No	No
Type of complications		Foreign body reaction to plate	Surgical Site Infection							

Select case presentations

Case One: Achilles Rupture Repair

**Figure 1:**
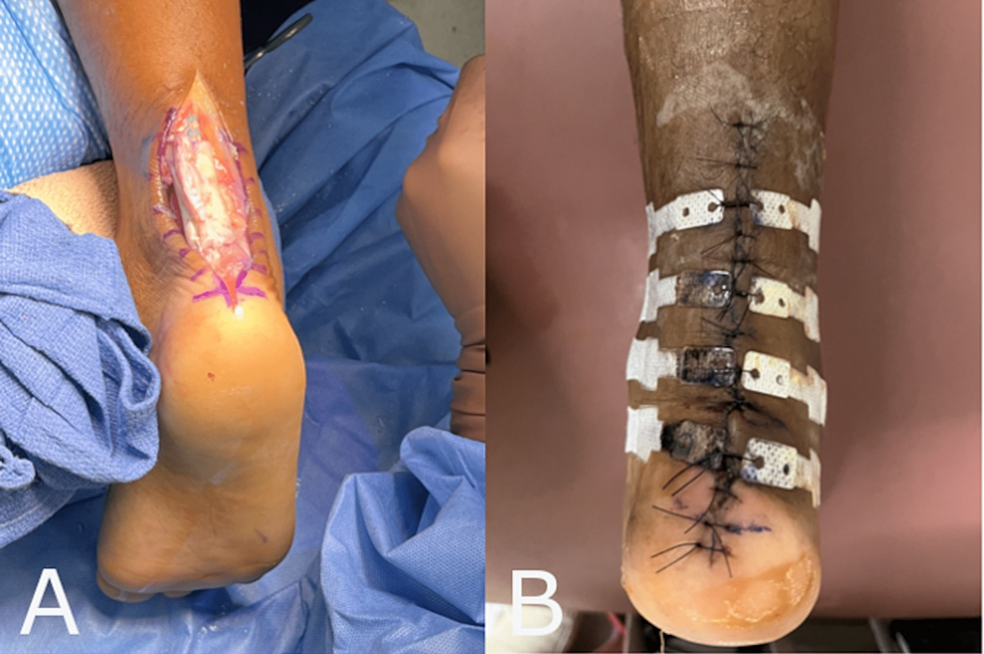
(A) The synthetic hybrid-scale fiber matrix applied over the Achilles tendon and sutured in place. (B) The surgical wound sutured closed.

A 38-year-old male presented with an Achilles rupture sustained while playing basketball. The patient did not have any notable co-morbidities. One week after initial presentation, the patient was taken to the operating room to perform an Achilles rupture repair. After the repair was performed, a 3 cm x 3 cm whole piece of synthetic hybrid-scale fiber matrix was applied to the surgical wound bed and sutured in place using resorbable sutures (Figure 1A). The wound was then sutured closed (Figure 1B). The surgical suture line was then dressed utilizing a nonadherent primary dressing soaked in betadine, 4 x 4 gauze, cotton undercast padding, and a 6-inch double elastic bandage. This patient went on to heal uneventfully. The physician elected to utilize the synthetic hybrid-scale fiber matrix in this case given the risk of delayed healing and wound dehiscence seen with Achilles rupture repairs.

Case Two: Trimalleolar Ankle Fracture Repair

**Figure 2:**
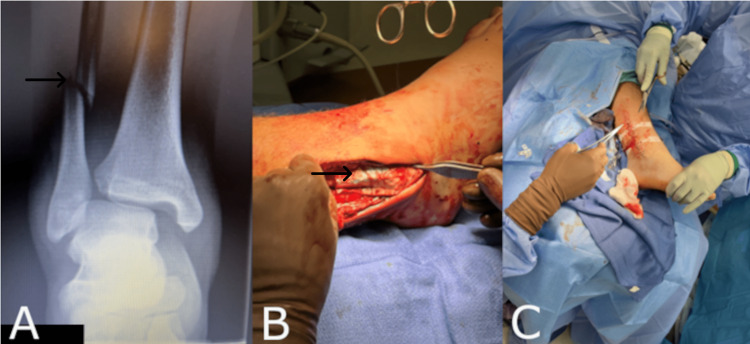
(A) Radiographic evidence of the fracture. (B) Application of the synthetic hybrid-scale fiber matrix to the surgical wound bed. (C) The surgical wound sutured closed.

A 15-year-old male with comorbidities including attention-deficit/hyperactivity disorder and obesity presented with a Trimalleolar ankle fracture sustained during a football game (Figure 2A). A week after the initial injury presentation, the patient was taken to the operating room. At the time of the procedure, the operating physician performed an open reduction and internal fixation (ORIF) of the Trimalleolar ankle fracture and syndesmosis, as well as a deltoid ligament repair. Extensive dissection was required for the proximal weber type C fracture reduction, as the closed reduction and percutaneous fixation were not successfully accomplished. The synthetic hybrid-scale fiber matrix was placed during the procedure to the risk of exposed hardware given the challenges of the procedure (Figure 2B). The surgical wound was sutured closed utilizing resorbable sutures and dressed with a nonadherent, betadine-soaked primary dressing, followed by gauze and a double elastic bandage. (Figure 2C). Three weeks postoperatively, the patient developed a foreign body reaction to an implanted plate and underwent an incision and drainage (I&D) procedure and was placed on oral antibiotics for two weeks. The synthetic hybrid-scale fiber matrix prevented the exposure of hardware resulting from the foreign body reaction and wound dehiscence. The wound healed uneventfully after the I&D.

Case Three: Bimalleolar Ankle Fracture Repair

**Figure 3:**
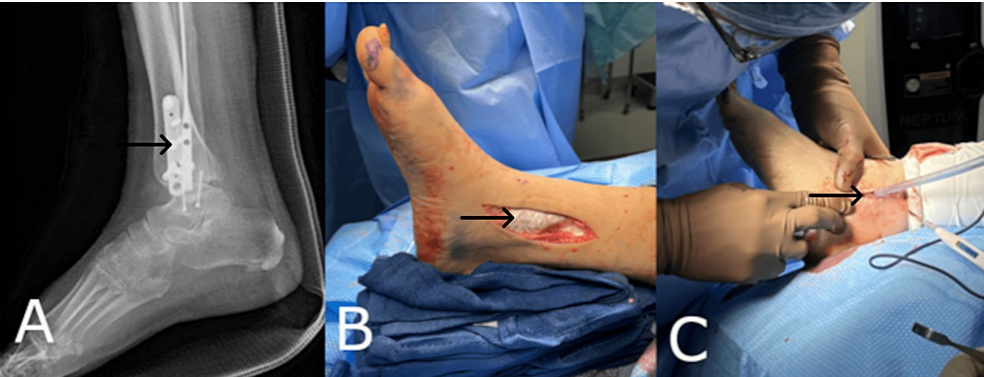
(A) Radiographic image of the ankle post open reduction and internal fixation procedure. (B) The synthetic hybrid-scale fiber matrix applied to the surgical wound bed and sutured in place. (C) The incision and drainage procedure performed after the development of the surgical site infection. The synthetic hybrid-scale fiber matrix prevented the exposure of deep hardware.

A 44-year-old female with a prior medical history including obesity, aortic stenosis, and end-stage renal disease presented with a bimalleolar ankle fracture. Ten days after the initial presentation of the injury, the patient was taken to the operating room to repair the injury. The surgeon preformed an ORIF of the bimalleolar ankle fracture (Figure 3A) and a total ankle arthroplasty with anatomic lateral ankle stabilization (ATLAS) utilizing the synthetic hybrid-scale fiber matrix (Figure 3B). The synthetic hybrid-scale fiber matrix was elected for this case due to the patient’s history of multiple postoperative infections and a history of wound dehiscence. The surgical wound was sutured closed using resorbable sutures. The wound was then dressed with a nonadherent betadine-soaked primary dressing, followed by 4 x 4 gauze, cotton undercast padding, and a double elastic bandage. The patient was instructed to use a splint. As expected from the patient’s medical history, she developed a surgical site infection after her initial procedure. In order to resolve the infection, an I&D procedure was performed in conjunction with a bone biopsy (Figure 3C). In this case, the application of the synthetic hybrid-scale fiber matrix during the initial surgery prevented the deep exposure of hardware and bone that would have been otherwise observed. After the I&D procedure, the patient was given intravenous antibiotics and went on to heal uneventfully.

Case Four: Bimalleolar Ankle Fracture Repair

**Figure 4:**
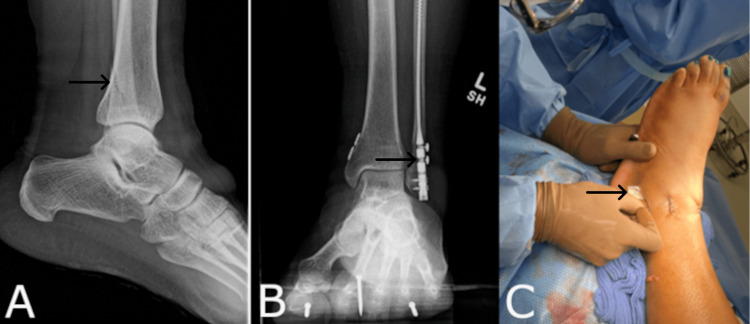
(A) Radiographic evidence of a bimalleolar ankle fracture. (B) Radiographic image of the ankle fracture after undergoing an open reduction internal fixation procedure. (C) The synthetic hybrid-scale fiber matrix being applied to the wound bed during surgical repair of the bimalleolar ankle fracture.

A 34-year-old woman with a prior history of cervical cancer presented with an ankle injury, which upon radiographic examination, was discovered to be a bimalleolar ankle fracture (Figure 4A). A week after the initial injury presentation, the patient was taken to the operating room for reconstructive surgery. The patient underwent an ORIF of the bimalleolar ankle fracture and syndesmosis (Figure 4B). The synthetic hybrid-scale fiber matrix was then placed in the surgical wound over the hardware, and the wound was sutured closed using resorbable sutures (Figure 4C). The surgeon elected to use the synthetic hybrid-scale fiber matrix due to the patient’s history of surgical site dehiscence. The wound was dressed in a nonadherent primary dressing, 4 x 4 gauze, cotton undercast padding, and an elastic bandage wrap. The patient was instructed to wear splint and went on to heal uneventfully.

Case Five: Heel Wound

**Figure 5:**
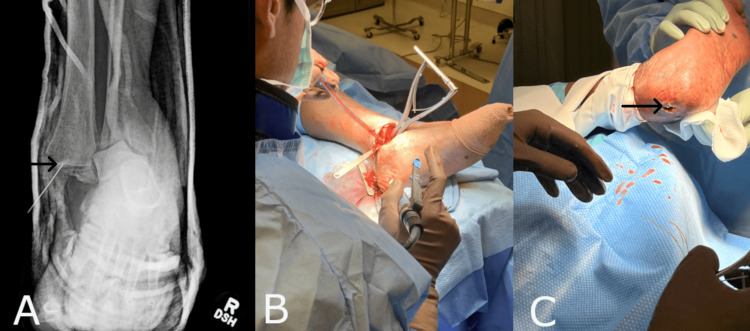
(A) Radiographic image of the ankle fracture. (B) Initial staged intervention and tibio-talo-calcaneal fusion procedure. (C) Heel wound during the I&D procedure I&D: Incision and drainage

A 76-year-old female with a past medical history of diabetes mellitus, hypertension, hyperlipidemia, cellulitis, and arthritis presented to her local emergency room with an open trimalleolar ankle fracture (Figure 5A). The patient underwent a staged intervention and a tibio-talo-calcaneal fusion (Figure 5B). Postoperatively, the patient developed a heel wound. The initial plan to address the wound was through conservative management of the wound by applying the synthetic hybrid-scale fiber matrix in the physician office setting. The patient decided to elect a more aggressive treatment plan instead of conservative grafting, and was ultimately brought back to the operating room for a formal I&D. During the I&D procedure, the synthetic hybrid-scale fiber matrix was placed in the heel wound in order to mitigate the risk of deep exposure and infection given the patient’s age and comorbidities (Figure 5C). The wound was dressed utilizing a non-adherent primary, 4 x 4 gauze, cotton undercast padding, and an elastic bandage wrap. The patient was instructed to utilize a surgical split. Six days postoperatively, the heel wound achieved full closure with no complications.

## Discussion

In this retrospective case series, the synthetic hybrid-scale fiber matrix was used to augment orthopedic reconstruction surgeries in high-risk procedures. The results of this retrospective case series demonstrated that 8 out of 10 patients went on to heal without any complications. In two of the cases where complications occurred (surgical site infection and foreign body reaction), the synthetic hybrid-scale fiber matrix prevented the exposure of hardware and bone. After these two wounds underwent an I&D procedure, they too went on to heal uneventfully. 

The results seen in this retrospective case series are comparable to those seen within this patient population. The incidence of surgical site infection in ankle fractures treated by ORIF is roughly 7.19% [3]. There are risk factors that increase the likelihood of an SSI occurring, such as obesity and prior surgical infections. A study conducted by Olsen et al. concluded that 14% of patients who underwent ORIF of an ankle fracture developed a surgical site infection [4]. Patients with a body mass index (BMI) greater than 30 developed SSIs more frequently, at a rate of about 20% [4]. One patient in this current retrospective case series developed an SSI infection and had risk factors that made the likelihood of this increased, such as obesity and previous surgical site infections. In some cases, SSIs require the removal of hardware, leading to prolonged recovery time and increased morbidity [13]. The patients in the case series, however, did not require the removal of hardware. The synthetic hybrid-scale fiber prevented the exposure of hardware, and the patients went on to recover without further complications. This was also seen in the patient who developed a foreign body reaction, in which exposure to hardware was prevented due in part to the synthetic hybrid-scale fiber matrix, and further complications were avoided. 

New surgical methods are needed to meet the needs of patients with known risk factors. The synthetic hybrid-scale fiber matrix is engineered to allow for cellular infiltration, re-vascularization, and differentiation [8]. Given the dysvascular nature of structures involved in orthopedic procedures such as bone and tendon, matrices that encourage tissue formation at these wound sites should be considered. Encouraging rapid re-vascularization to these surgical sites is vital to reduce risk of delayed healing, as prior research has suggested that weak blood flow to the lower extremities can increase risk of complications and delayed healing [14]. The porosity of the synthetic hybrid-scale fiber matrix increases as it resorbs, and this continuous increase in the porosity supports increasing tissue formation and vascularization [8]. 

Several previous studies have assessed the use of the synthetic hybrid-scale fiber matrix in the treatment of lower extremity surgical procedures. A separate retrospective case series of 12 patients who underwent peroneal tendon repairs augmented by the synthetic hybrid-scale fiber matrix was conducted at a single site [15]. The patients had no complications postoperatively, and 11 out of 12 patients reported little to no pain following surgical treatment [15]. Pain levels were assessed utilizing the visual analogue scale (VAS). At six months postoperatively, the average pain score was 1.6/10 and many patients had returned to normal activities [15]. Other published case series have assessed the utility of the synthetic hybrid-scale fiber matrix in the treatment of chronic lower extremity wounds. In a retrospective case series conducted by Barton and Abicht, 23 lower extremity wounds including transmetatarsal amputations, venous ulcers, and diabetic foot ulcers were treated with the synthetic hybrid-scale fiber matrix [12]. Out of these 23 wounds, 96% achieved total wound closure in an average of 95 days [12]. Of note, both aforementioned studies are retrospective in nature, and therefore were subject to recall bias and lack of a control group. Similar results were seen in a prospective, single-arm study of diabetic foot ulcers, in which 75% of wounds achieved closure within 12 weeks of treatment with the synthetic hybrid-scale fiber matrix [10]. The positive results observed with the synthetic hybrid-scale fiber matrix in the treatment of lower extremity chronic wounds and injuries indicate that the matrix may be a viable treatment option in this clinical setting.

There are limitations with this retrospective case series. This was a small retrospective study conducted by a single investigator at a single site. As with all retrospective studies, this study is subject to design flaws such as availability of medical records and recall bias [16]. In addition to this, this case series involved multiple surgical procedures as opposed to focusing on one clinical use case, which can complicate the ability to draw conclusions that are generalizable to a specific surgical procedure. There was also no control group or standardized method for applying the synthetic matrix. Further studies with a larger cohort of patients stratified by procedure should be considered to investigate these results, including an assessment of postoperative pain and quality of the healed wound. 

## Conclusions

Orthopedic surgical repairs of the leg and ankle are not without risk, especially in patients with multiple comorbidities or those with a history of poor wound healing. New modalities are needed to mitigate risk amongst this population. A synthetic hybrid-scale fiber matrix may provide an option for improving wound healing.

This retrospective case series demonstrates successful application of the synthetic hybrid-scale fiber matrix in the setting of orthopedic surgical repair. The results observed indicate that the synthetic hybrid-scale fiber matrix could be utilized to promote healing and may be considered as a treatment option in at risk reconstructive procedures of the lower extremity.
